# ASO Author Reflections: Complex General Surgical Oncology Fellowship Training: Variation in Outcomes of Graduates Performing Hepatopancreatic Surgery by Graduating Fellowship Program

**DOI:** 10.1245/s10434-025-17645-7

**Published:** 2025-06-09

**Authors:** Diamantis I. Tsilimigras, Timothy M. Pawlik

**Affiliations:** Division of Surgical Oncology, Department of Surgery, The Ohio State University Wexner Medical Center and James Comprehensive Cancer Center, Columbus, OH USA

## Past

Hepatopancreatic (HP) surgery is a technically complex field requiring specialized training prior to independent practice.^1–3^ In the USA, the vast majority of HP surgeries are currently performed by graduates of complex general surgical oncology (CGSO) fellowship programs.^4^ While minimum case requirements for graduation are universal across all CGSO programs, certain programs offer a more robust/heavy training in HP surgery than other programs. To date, real-world data evaluating outcomes of CGSO graduates performing HP surgery have been lacking. As such, we sought to assess variations in outcomes after HP surgery across surgeons trained at different CGSO programs.^5^

## Present

Between 2016 and 2021, a total of 9954 Medicare beneficiaries underwent HP surgery for cancer (pancreatectomy: 76.0%; hepatectomy: 24.0%); these cases were performed by a total of 609 CGSO fellowship-trained surgeons. Notably, nearly half of HP operations were performed by graduates of just two CGSO programs (47.9%), whereas more than 90% of operations were performed by graduates of only 15 out of 42 total CGSO programs. After adjusting for patient- (e.g., age, comorbidities, etc.), procedure- (e.g., case complexity, elective/urgent, etc.), hospital- (e.g., rurality, teaching status, nurse-to-bed ratio, etc.), and surgeon- (e.g., surgeon sex, volume, years in practice, etc.) level characteristics, marked variation in the adjusted probability of serious complications and 90-day mortality following HP surgery was noted by graduating CGSO fellowship program. For example, the adjusted probability of severe complications following HP surgery among graduates of CGSO program #10 was 9.7% versus 21.7% (%difference 12%) among graduates of program #13. Large variations in 90-day mortality were similarly observed when assessing outcomes across graduates of different CGSO programs. These differences persisted across all surgeons as well as among early stage career surgical oncologists. Despite these variations, the adjusted risk of serious complications after HP surgery decreased during the study period from 2016 to 2021.

## Future

To our knowledge, this is the first study to report real-world outcomes among graduates of different CGSO programs. The exact reasons behind the substantial variations in outcomes after HP surgery by CGSO program warrant further investigation. Although training data were limited to the program these surgeons graduated from, future studies should explore training specific data including operative case volume during training and their association with post-graduation outcomes. Dual certification in both CGSO and HPB surgery may enhance operative exposure in HP surgery during CGSO fellowship. Future studies should assess whether additional HPB-focused training during CGSO fellowship impacts outcomes of CGSO-trained surgeons performing HP surgery after fellowship graduation. Furthermore, further efforts need to be made to standardize HP training across different programs and optimize HP-specific curriculum within CGSO fellowship pathway to ensure high-quality, consistent training nationwide.
